# Lectures on the Morbid Anatomy of the Serous and Mucous Membranes

**Published:** 1837-07

**Authors:** 


### Art. II.

*Lectures on the Morbid Anatomy of the Serous and Mucous Membranes.* By Thomas Hodgkin, m.d., Demonstrator of Morbid Anatomy, and Curator of the Museum, at Guy's Hospital, &c. Vol. I. *On the Serous Membranes, Parasitic Animals, Malignant Adventitious Structures, and the Indications afforded by Colour.—London,* 1836. 8vo. pp. 402.

The present volume contains twelve lectures. The first is introductory; the next five are devoted to the subject of the Serous Membranes; the seventh, to the consideration of Parasitic Animals; the eighth, to adventitious Serous Membranes; the ninth, tenth, and twelfth, to Malignant Diseases; and the eleventh is on the Colours of the Animal Tissues.

The importance of the subjects embraced by these lectures, and the reputation which Dr. Hodgkin has justly acquired as a zealous cultivator of pathological anatomy, are sufficient to claim the earnest attention of every reader to the work before us. Any lengthened prefatory observations, therefore, would here be out of place; and we proceed at once to the consideration of the Introductory Lecture, which, as explanatory of the views of the author, is entitled to some preliminary notice. This lecture comprises, in addition to some judicious remarks upon the importance of morbid anatomy, and the expediency of examining all cases of fatal disease which may fall under our notice, an interesting though brief outline of the progress of this part of medical knowledge, from the earliest periods of medical history down to the present time.

There are three modes in which the phenomena of pathological anatomy may be considered and arranged; and each has its peculiar advantages. The first of these is founded upon the anatomy of regions, and is that followed by Dr. Baillie; the second is based upon the principles of general anatomy, which, as will at once appear from the title of the work, is the method adopted in the series of lectures before us; the third, and certainly, however the convenience of teaching may for a time induce a preference of one of the preceding methods, the most philosophical and scientific, is that pursued by Dr. Carswell, in his splendid and truly valuable "Illustrations of the Elementary Forms of Disease." That there are certain disadvantages, even for the purposes of tuition, attending the plan followed in the present treatise, Dr. Hodgkin is himself apparently aware, as he has so far followed Dr. Carswell as to consider some morbid degenerations separately from the tissues in which they occur; for instance, Melanosis, Carcinoma, and other forms of malignant disease.

The arrangement here followed is the same as that adopted in the classification of the morbid preparations belonging to the museum of Guy's Hospital.

"Deviations from the normal state; consisting—

"1. In deficiency,

*a.* The result of suspended development.

*b.* Loss sustained.

"2. In excess.

"3. In form.
"4. In appearances which may be regarded as the result of ordinary inflammation.
"5. In appearances which are the result of scrofula.
"6. In appearances which are the result of diseases called malignant, or resembling them in structure.
"7. In hydatids in the particular organ.
"8. In the effects of accidental injury." (P. 16.)

Of the lectures more especially devoted to the Serous Membranes, the second relates to the serous membranes generally; the third, to the arachnoid membrane; the fourth, to the pericardium; the fifth, to the pleura; and the sixth, to the peritoneum, tunica vaginalis, and bursæ.

Previously to describing the morbid alterations of tissue, or other degenerations to which the serous membranes are liable, the author gives a short summary of the characters presented by them in the state of health. The observations upon this point, though perhaps not strictly relevant to the professed subject of the lectures, might have been extended with advantage, especially with reference to the minute structure of the membranes, developed by the microscope. Dr. Hodgkin advocates, as our readers know, the opinion that the ultimate structure of the primitive tissues is fibrous, and not globular, or spheroidal; and he repeats the observation here with respect to the serous membranes in particular.

"The ultimate structure of these membranes, like that of the cellular tissue, has been regarded by most minute observers, both British and foreign, as consisting of extremely minute fibrillæ, so combined as to form lamellæ. By many modern physiologists, French and German, as well as English, these fibrillæ are believed to be composed, in their ultimate analysis, of globules, arranged like strings of beads. This opinion, I am fully convinced, has its sole foundation in an optical illusion. My friend, Joseph Lister, who has carried the powers of the microscope far beyond anything to which they had previously attained, has very minutely examined this, as well as most of the other animal tissues. He and myself have spent hours in the most careful examination of the subject, and not the smallest doubt is left on our minds as to the absolute fallacy of the globular theory." (P. 26.)

Notwithstanding this statement, we must remark that, among those who differ from Dr. Hodgkin and Mr. Lister, are those admirable observers, Hewson, Bauer, Prevost and Dumas, Dutrochet, Meckel, and others. Meckel, as Dr. Hodgkin states, considers the serous membranes as consisting of a coherent, homogeneous, viscid, amorphous substance, condensed in broad layers; but this description is said to apply only to recent and imperfectly formed membranes. Assuming, however, the microscopical observations of Mr. Lister to be the most accurate hitherto made, and in themselves free from those optical illusions which are presumed to have misled others, we do not see how the globular theory is set aside by "*à priori* reasoning," any more than the fibrous, or any other which might be brought forward. "The extremely delicate textures," says Dr. Hodgkin, and the remark is correct, "of which animals of the most minute microscopic size are formed, precludes the possibility of the ultimate structure being visible to our eyes, however aided by microscopes. There are animalcules of such minute size, that their entire bulk is not equal in volume to that ascribed by the advocates of the globular theory to one of their integrant or primitive molecules."

(P. 26.) But, as we have already remarked, however this mode of reasoning may militate against the reception of the globular theory, it equally militates against all others relating to the ultimate elementary composition of this and other tissues. The real question is, not what is the ultimate structure, but what is that structure which our means of investigation have made known to us as the most minute and elementary hitherto developed? Mr. Lister and Dr. Hodgkin assert that it is the fibrous. Other observers, of acknowledged talent, and of competent skill in the use of the microscope, assert that they have gone further; that they have succeeded in developing a globular or spheroidal structure in the minute fibrillæ, into which the lamellæ or plates of the serous membrane are primarily resolved. Unless we are to admit that these last have been misled by optical illusion, which we confess ourselves unwilling to do,—as, in that case, we must throw the same doubt over all the investigations which have been made, and may yet be made, into elementary structure,—we must, in candour, still hold the question undecided, until repeated observations by both parties shall have further cleared up the obscurity in which the subject is involved.

The question of the vascularity of serous membranes is also one upon which there is considerable difference of opinion, and, intimately connected as it is with the subject of inflammation, we should have been glad to have seen it treated somewhat more at length. Rudolphi and others maintain that vessels do not exist within the texture of the proper serous tissue, although they ramify extensively in, and perhaps over, the reticular texture immediately beneath. Dr. Hodgkin is disposed to think, from the elementary structure of the tissue, that the fibres and laminæ of which it is composed may admit of the passage and ramification of vessels through and within its substance. As far as we have been able to observe, we are inclined to agree with M. Gendrin* that, with some few exceptions, as in the pleura towards the summit of the lungs, and in some portions of the peritoneum, the serous membranes are not vascular; the minute vessels ramifying in the cellular or subserous tissue, to which these membranes are more or less intimately united, pouring out their fluids either upon the free surface, through the medium of minute exhalent branches passing directly through the tissue, or otherwise into the subserous reticular tissue, whence the effused fluid rapidly transudes through the minute pores of the proper serous tissue by a process analogous to imbibition.

It has been doubted whether the serous tissues are supplied with nerves; and a proof of their deficiency has been sought for in the fact, that, in the state of health, they are endued with little or no sensibility. On the other hand, their extreme sensibility in a state of disease has been considered to afford strong grounds to believe that they are not destitute of nervous fibrils, though hitherto none have been detected in their texture. Without entering into this question, we may observe that the existence of pain, arising from injury of parts of such extreme tenuity, and so intimately connected with other organs, can scarcely be considered as an argument in favour of their possessing nerves, as it is almost impossible to produce irritation, mechanical, chemical, or vital, of such a

* Histoire Anatomique des Inflammations, t. i. p. 47.

texture, without involving other organs and tissues, closely, and indeed inseparably, connected with it. The tunica vaginalis is extremely sensible to pain when injected in the operation for hydrocele; but this pain does not usually occur until a few seconds after the injection of the stimulating fluid, and may equally arise from the sensibility developed in the parts beneath by the absorption or the imbibition of a portion of the fluid.

Passing over those alterations of tissue which consist either in its deficiency, in its excess, or in irregularity of form, which are for the most part connected with and dependent upon corresponding deviations from the normal state in the subjacent viscera, we proceed to examine those morbid changes in the serous membranes which may be regarded as the results of inflammation, and the diseased processes connected therewith. Alluding to the conflicting opinions of authors respecting the nature of inflammation, Dr. Hodgkin himself declines attempting a definition of what he understands by this term, leaving his opinions to be gathered from the facts subsequently brought forward. We are not inclined to question the propriety of this proceeding, as, however convenient it may be to have a brief summary of the essential characters of any object which may be brought before us, it cannot be denied that the real use of such compendious definitions has been too often mistaken; and what was merely intended to facilitate the acquisition of important knowledge has not unfrequently been substituted for that knowledge itself. The celebrated definition, "*rubor et tumor cum calore et dolore*," has been at least the cause of as much superficial and erroneous thinking, as it has been of advantage to the progress of the student. Inflammation is a particular mode of action in the tissues and organs of the animal body, and a knowledge of it can only be acquired from its effects. The phenomena of inflammation presented to us in the serous membranes differ from those which the same principle, or action, or deviation from the physiological state, presents in any other tissues or organs; any general definition or abstract idea of the term, unless we had arrived further in the elimination of the laws of health as well as disease than our present knowledge seems capable of carrying us, would only therefore lead into error.

A suppression of the usual secretion would appear to be one of the first appreciable effects of inflammation of serous membranes; and, from the analogy of the mucous membranes, we are inclined to think this of more frequent occurrence than is commonly supposed: it is sufficiently obvious, however, that this state can seldom come under the notice of the pathologist, although it has been observed. The increase of secretion, or rather, we would say, the pouring out from the exhalents communicating with the distended capillaries of the subserous tissue, of the more fluid parts of the blood, is, on the other hand, so frequent as to be considered one of the usual terminations of inflammation in those tissues. Some authors have thought that the effused fluids may be simply augmented in quantity, preserving all the qualities of the ordinary serous secretion. Where this is the case, if it really does occur, we should be disposed to doubt its being an effect of inflammation. The fluid which moistens the unattached surfaces of the serous membranes, and which is presumed to be a secretion from them, is by no means identical with the serum of the blood; for the most superficial examination will show that, in each several variety which the serous tissue presents, the secretion differs in its sensible qualities. The secretion furnished by the arachnoid is, as Dr. Hodgkin remarks, the most aqueous of the serous secretions; the secretions from the peritoneum, pleura, and pericardium, appear, independently of any morbid alteration, to be more charged with animal matter; those of the synovial bursæ and of the capsules are still more viscid; while in serous cysts,—an adventitious production, it is true, but so strictly analogous to serous membranes as to admit of their being adduced here to strengthen the preceding observations,—we find every variety of secretion, from a perfectly clear, limpid, aqueous fluid, up to mucus not less clear and perfect than any produced by the Schneiderian membrane or the follicles of Naboth. (P. 29.) Dr. Hodgkin makes use of the preceding facts to show the gradual transition from serous to mucous tissues. Those cases in which the fluid contained in serous cavities most resembles the natural secretion of the respective cavities are passive or atonic dropsies, which may be supposed to depend upon a diminished power of the absorbent system, or obstruction to the course of the blood. These, however, are of much rarer occurrence than is commonly supposed, even at this time, when the inflammatory origin of many forms of dropsical effusion has been so fully recognized. That the fluid poured out by the exhalents, in certain cases of inflamed serous membrane, is derived at once from the blood, independently of any secreting process, is rendered more evident by an examination of the varieties which these effusions present. Sometimes they are perfectly limpid and of a pale straw colour, and strictly analogous to the serum of the blood. Sometimes they are more or less tinged red, as if from the admixture of blood; the vessels having, in such instances, permitted the passage of some of the red particles of that fluid; while, in other cases, blood, more or less pure, is found to have escaped from its vessels, without the rupture of any large branch to account for its presence in the serous cavity.

We are far from wishing to attempt a refinement in our pathological distinctions which is not borne out by well-ascertained facts, but we cannot but think that, in such cases as those to which allusion has just been made, the inflammation is seated in the subserous cellular tissue, without implicating the serous membrane itself. We are led to this conclusion, partly from a consideration of the phenomena occurring in inflammations of the membranes of the brain and those covering the abdominal viscera, to which we shall have occasion again to refer, and partly from observing that inflammation unquestionably affecting the serous tissue is marked by the occurrence of other morbid changes, differing considerably in their character. Dr. Hodgkin has not attempted to draw this distinction, but the following remarks appear to justify our conclusion:

"When these appearances (turbidity of the serous effusion and false membranes,) are not observed in persons who have died of suspected pleurisy, inflammation of the muscles or cellular membrane must, in most instances, have been mistaken for it. Yet, according to the author to whom I have already alluded, (Villermé,) an individual may die, after having exhibited all the marks of pleuritic inflammation, and little if any trace of it may be found on inspection. He accounts for this anomaly by supposing that the red blood leaves the capillaries, unless they had admitted it for some time before death. Such cases, I have no doubt, are extremely rare. From my own experience, I can cite none in which the inflammation, however recent, had not left some traces of its existence. If the inflammation have been very rapid and intense, and the patient have died on the second or third day, it is very possible that no false membrane may have been formed; but we find an effusion of serum, which, in the majority of cases, is sanguinolent or puriform. In a few instances I have noticed an effusion, nearly or quite clear and transparent, but possessing the power of coagulating on its removal from the body, after the manner of blood, but in a much feebler degree. The serous membrane affording this effusion is more manifestly vascular than in its physiological state; and this increased vascularity, instead of being uniformly diffused, more frequently produces the appearance of numerous, very minute, closely placed, and irregular red points; and is generally accompanied by small ecchymosed spots on the cellular membrane, situated behind the serous." (P. 37.)

The appearance of the serous tissue here described, and which must be carefully distinguished from the ramiform vascularity occupying the subserous cellular tissue, is precisely that which we consider to be among the earlier changes produced by inflammation seated in the serous membrane itself. In such a case we have observed the red points to assume the form of minute stars, at the same time that patches or shreds of lymph occupied portions of the free surface of the membrane. These cases are necessarily of rare occurrence, as death, in this country at least, does not commonly occur in so early a stage of inflamed serous membrane. Accordingly, Dr. Hodgkin refers to only four or five instances, as observed by Villermé, Dupuytren, and himself; and it is perhaps worthy of remark, that in each of these the pleura was the serous membrane affected. The case to which we have alluded above, which was also seated in that membrane, we look upon to afford an example of the transition from the state described by Dr. Hodgkin, to those later stages of frequent occurrence, in which there is effusion, more or less abundant, of turbid or sero-purulent fluid, with the formation of false membranes upon the serous surfaces.

The mode of formation of false membranes constitutes an enquiry of great importance, in the endeavour to trace out the phenomena of inflammation in these tissues; and, accordingly, the author has entered into this question at some length in his second lecture. After stating the views of Dupuytren and Villermé, he proceeds to give the conclusions at which he has himself arrived, from researches undertaken with the object of determining the correctness of these views. The first stage in the formation of false membranes, according to Villermé, is a pulpy state of the serous tissue, with an appearance of villi occurring at spots in which the inflammation is most intense; and these villosities, which are very short, generally of a dead white colour, delicate in structure, and easily removed, increasing and combining together, are conceived to constitute the false membrane, at the same time that some of the villi, becoming detached by the friction of the opposing surface of the serous membranes, are suspended in the effused fluid, rendering it turbulent and flocculent. Whenever, during a morbid process, interstitial effusion or deposition takes place, a certain development of the natural structure of the part would seem to arise; although, when the progress of such effusion or deposition is rapid, as in parts where considerable laxity of tissue prevails, the structure soon becomes so far effaced as to be with difficulty, if at all, recognizable. It may be doubted, therefore, whether these villosities of M. Villermé, at least in their earlier states, are any thing more than the natural structure of the membrane, more highly developed by the morbid actions going on therein; since it has been remarked by Gendrin, that the smooth surface of serous membranes is covered with villi, as may be readily perceived with a high magnifying power, though only sensible to the unassisted eye after some days' maceration. If these suggestions be admitted, the inference may be drawn that this state of the serous surface is that which immediately precedes, and is probably influential in the pouring out or the secretion of those parts of the blood which, from their plastic nature, are disposed to assume the concrete form; while the laminæ or films thus deposited upon the free surface of the inflamed membrane from the exudation of this plastic fluid will be of various degrees of tenuity and tenacity, according to the variations in the nature of the lymph effused, which again will vary with the intensity of the inflammatory action, the constitution of the patient, the state of the blood, and other conditions of the organization, to which we need not here advert.

The preceding statements are not inconsistent with, but on the contrary naturally lead to, the opinion of Dr. Hodgkin, which, as he observes, is "in some points little more than a return to the doctrine of John Hunter, though not borrowed from him."

"I find myself perfectly in accordance with this great master of his profession, in regarding the formation of the tender diaphanous fibres, composed of the plastic part of the effusion, (the coagulable lymph of John Hunter,) as the result of a process of the same nature with the coagulation of the blood. Hence, from the first they are continuous and homogeneous, even whilst they can scarcely be said to be solid; a state which they attain by the separation of the serum, rather than by the aggregation of floating particles or villi. Notwithstanding their tenuity, and soft and tender texture, they often form bags or pouches capable of retaining the serum. This fact appears to me to militate strongly against the idea entertained by Villermé, and sanctioned by no less an authority than that of Beclard, that the form of membrane or film is produced by the aggregation of an infinite number of minute flocculi. Were this the case, we should not only find most recent membranes, to a certain degree, cribriform, and incapable of retaining fluid, but, as Villermé seems to admit, they would be more or less opaque. To obviate this objection, he appears to have considered the transparent membranes as farther advanced than he is warranted in doing." (P. 45.)

False membranes of this description are of a loose cellular texture, and in appearance transparent and filmy. They constitute many of those membranous adhesions so frequently found to exist between the folds of the peritoneum and pleura, some of which are easily broken down, while others, on the contrary, are so firm as to be altogether inseparable from the parts which are united by them.

The changes of which we have hitherto spoken would seem to result, in a great measure, from the separation of the more simple elements of the blood, by a process, in some points of view, analogous to filtration. Those of which we are now to speak are more strictly the products of vitiated secretion, originating however in inflammation, and combined with the effects resulting from that process. In cases of this description there appears to be often some peculiarity of constitution in the patient, or of the external circumstances in which he is placed, modifying the character and interfering with the ordinary progress of the inflammation. Where these conditions exist, or when the inflammation has been protracted from various causes, the effused fluids are found to be turbid opaque, of a yellowish white colour, and more or less viscid; the adhesions, if any exist, loose and readily broken down; the serous membrane covered in patches of greater or less extent, with shreds or laminæ, which are loose in texture, opaque, and of a pale yellow colour, occasionally with a reddish tinge, and readily detached from its surface. Every variety, however, may be observed between these loose, flocculent, scarcely adherent patches, and the firmest and most completely organized membranous expansion. We shall have occasion to return to this subject when considering modifications of these processes as they occur in the several forms of serous membrane; but we recommend a careful perusal of Dr. Hodgkin's excellent observations in his second lecture, in which the leading points are well brought forward.

There is one other point connected with false membranes in general, which we cannot pass without some brief notice: we allude to the manner in which they become organized. Without entering into the various hypotheses proposed upon this subject, we shall content ourselves with quoting the opinion entertained by our author.

"My own opinion is, that, at the inflamed part, the minute blood-vessels not merely become distended, but that their delicate parietes, and the structure through which they ramify, become softened, and, yielding to the pressure of the blood in the distended vessels, give way at numerous minute points. This I believe to be the explanation of the fact, that, when we raise an adherent false membrane, of a few days' standing, we find that the original serous membrane beneath has lost somewhat of its polish, and presents numerous minute bloody points. The very small quantity of blood thus escaping from its vessels does not diffuse itself, but is received into the soft substance of the false membrane, which accordingly exhibits numerous bloody points on the surface which has been detached from the serous membrane. These spots, which are at first irregular, afterwards have a dendritic appearance, and, extending in length, they become vessels. These vessels, being feebly supported, are distended and larger than those in the original structure from which they proceed; hence the redness of newly organized false membranes. At a subsequent period these vessels contract, and may become nearly or quite invisible." (P. 50.)

This explanation, though not perfectly satisfactory, is at least equal to any other which has hitherto been proposed, and certainly far superior to the mechanical hypotheses advocated by Sir Everard Home: we must not, however, omit to state that some facts have been recorded which it would be difficult to reconcile with these views. Among others, we may refer to a case of acute arachnitis, reported by M. Gendrin, in which a false membrane, almost as dense as the dura mater, was found covering the upper part of the left hemisphere of the cerebrum with the ramifications of vessels developed in its substance. "This membrane," says M. Gendrin, "adhered slightly to the cranian arachnoid, and not to the cerebral hemisphere upon which it was applied; but, at the place where the adhesion was found, the arachnoid appeared to be healthy, and the origin of the vessels belonging to the secretion was not discovered."

The results of inflammation in the serous membranes of the brain and its appendages are ably discussed by Dr. Hodgkin, in his third lecture. He considers the arachnoid to consist of four portions: first, that which is external to the brain; second, that which lines the ventricles; third, that which belongs to the plexus choroides; and, fourth, that of the spinal cord. In this arrangement we shall follow the author in the observations which we have to make, as the divisions adopted by Foville, although they appear to us better calculated to exhibit the relations existing between the several morbid changes and the pathological conditions giving rise to them, are scarcely so well fitted to show the relations which these morbid changes bear to each other.

We are inclined to doubt whether the frequency of occurrence of the more decided products of inflammation (sero-purulent effusion and false membrane,) between the free surfaces of the arachnoid external to the brain, is not somewhat under-rated by the author. When we find so practical a pathologist as Dr. Hodgkin stating that he had "long been fruitlessly seeking for an example, not traumatic, of this form of arachnitis," we cannot but suspect that this extreme infrequency may be attributed, in part at least, to some peculiar circumstances of locality or situation in which he is placed. At the same time, it is sufficiently evident that such appearances must be of rare occurrence, since the inflammation, in the greater number of cases, would necessarily destroy the patient, by the extent of the serous effusion and other changes induced before it attained the stage in which lymphous exudation or vitiated secretion could arise. We have, however, ourselves met with a case in which there was considerable effusion of lympho-purulent matter at the base of the brain, with adhesions between the surfaces of the arachnoid, in the person of a female dying of fever. Dr. Hodgkin refers to a case by Rostan, and to several others reported by Foville and Guersent. In a remarkable case in which the serous membranes generally were inflamed, narrated by Gendrin,* the arachnoid of the convexity of the brain and of its base was red, injected, thickened, and covered by a moderately thick purulent layer, and the pia mater was infiltrated with sanguinolent serosity; and the same author reports other examples,† in which there were inflammatory exudations, of various degrees of colour, substance, and density, more or less adherent to one or other of the free surfaces of the arachnoidal sac.

The products of inflammation in the loose cellular texture situated between the arachnoid membrane and the surface of the brain, are of much more frequent occurrence than those found in the cavity of the arachnoid. These products are, effusions of serous fluid, of various degrees of tenuity and transparence, and changes taking place in the subserous cellular structure itself. M. Foville's graphic description of the earliest stage of meningitis is quoted by the author; and most of our readers, as well as ourselves,—notwithstanding that it is rare for the disease to prove fatal in the first stage,—can, doubtless, testify to the accuracy and fidelity of the account. The exudations beneath the cerebral arachnoid have presented very different characters in different examples of fatal arachnitis which have fallen under our notice, and we think that Dr. Hodgkin has scarcely devoted sufficient attention to this part of his subject.

> "Though, after the most active symptoms of arachnitis," he observes, "we often find nothing but serum infiltrated behind the arachnoid, we at times meet with a coagulable effusion, and, rather more frequently, with one that is puriform." (P. 73.)

* Histoire Anatomique des Inflammations, t. i. p. 75.

† Op. cit. Cases 11, 12, 24, 25, 26, 28, 29.

. . . . "A large quantity of serum is sometimes found beneath the arachnoid covering the brain, in cases in which, if inflammation had been the cause, it must have been of a very chronic character. The fluid may be found collected at a particular part, and the form of the brain may be greatly modified by its presence. In one case which I saw in the Salpêtrière, several ounces of serum were collected beneath the arachnoid, covering the anterior lobes of both hemispheres." (P. 76.)

These extracts, which, with the exception of a brief allusion to some instances where the inflammatory exudation is puriform, and not unfrequently of a greenish colour, comprise nearly all that is stated by the author on this point, give but an imperfect view of what we have had occasion to observe. We find here no mention of the numerous gradations which the effused or secreted fluid presents in respect of viscidity; of its occasional gelatinous appearance, a pathological condition which we believe to be of not very uncommon occurrence; of its occasional sanguinolent character, and of the pulpy dissolved state to which the pia mater is in such instances sometimes reduced. This gelatiniform state of the serous secretion we are disposed to think is commonly the result of the more chronic forms of meningitis, and is sometimes found to proceed to great extent before the patient falls a victim to the disease. A case is related by Neumann,* in which the arachnoid membrane could not be distinguished, being apparently transformed into a gelatiniform mass, which occupied its situation on the surface of the cerebral hemispheres.

The layers of cellular tissue constituting the pia mater are not unfrequently thickened and opaque where the infiltrations of serous fluid are abundant, and similar appearances are found in the arachnoid. The morbid alterations in the structure of the arachnoid itself, however, need not detain us long: the principal are loss of polish and smoothness of its free surface, and opacity of a pearly or opalescent character, with or without apparent thickening of its substance. The arachnoid has been said to be so much altered in some rare cases of chronic meningitis as to equal nearly the dura mater in thickness, and even in tenacity; but there is much reason to doubt whether, in such instances, the superior layers of the tissue of the pia mater may not have been the principal seat of the interstitial deposition.

The arachnoid lining the ventricles is less frequently altered in its characters, although effusion of limpid fluid into these cavities is one of the most common appearances discovered in morbid conditions of the brain. The effused fluid is occasionally sanguinolent, and sometimes puriform, but is rarely found to contain lymphy particles. Dr. Hodgkin alludes also to a peculiar roughness of the free surface of the membrane which is occasionally observed.

The section upon the Arachnoid of the Spinal Cord, although very nteresting, presents nothing which calls for especial notice; and we proceed therefore to take a cursory view of those morbid appearances which are the results of inflammation in the serous cavities of the heart, chest, and abdomen.

The principal products of inflammation in the pericardium are the following:—Vascularity of the membrane, more or less fluid in the sac,

* Archives Gén. de Médecine, tome 6.

which may be limpid and apparently but little altered from the natural secretion, sanguinolent, opaque and puriform, or turbid; close and intimate, in some cases inseparable adhesions between the two surfaces, with great thickening and interstitial deposition; loose membranous adhesions readily giving way on the application of a slight force; bands of false membrane, of more or less firmness and extent; and a peculiar, shaggy, ragged coating, arising from the deposition of shreds of false membrane, in a loose flocculent form, on the free surface of the natural tunic.

Vascular injection of the pericardium is rarely observed to assume the form of striæ, and the redness of the membrane in portions recently inflamed is found chiefly to occupy patches of greater or less extent, uniformly tinged, and sometimes having numerous points of a deeper red scattered over their surface. The serous secretion is very commonly found considerably increased in quantity; and this appearance would seem to depend upon various causes. Not unfrequently it is a phenomenon purely cadaveric; sometimes it may possibly be a passive effusion, of the nature of atonic dropsy. In such cases the effused fluid has been described as abundant, limpid, and of a pale yellow colour, but little altered in its sensible characters from the ordinary secretion. Fluid of this description is occasionally found in persons labouring under some of the forms of dropsy, and also in cases of obstructed circulation, from whatever cause: we believe, however, with Laennec, Bertin, and Dr. Hodgkin, that the purely atonic form of effusion, constituting the true hydrops pericardii, or hydropericardium, is by no means a common affection; many of the cases which have been supposed to be instances of it being the result of inflammation of the serous tissue itself, or of the struggle which precedes the termination of life. The effusion is sometimes exceedingly copious, and Dr. Hodgkin refers to some cases of this description, among others to the extraordinary one mentioned by Corvisart, and since so repeatedly quoted by authors, in which the quantity of fluid amounted to eight pounds. Laennec has recognized, as a distinct form of pericarditis, the cases in which the effusion is sanguinolent, or accompanied by an admixture of blood. Dr. Hodgkin, however, says, "a slight admixture of blood not unfrequently accompanies many preternaturally increased exhalations and secretions, whether inflammatory or not: it may be seen in the perspiration from the skin, the mucus from the nose, the urine, the secretion from the testes, and in hydropic effusions, as well as in inflammations of the serous and mucous membranes." (P. 95.) This opinion must, we think, be received with some limitation; for, except in instances of what may be termed the hemorrhagic constitution, —in purpura and in scorbutus, for example,—we doubt much whether the secreted fluids are often, if at all, found sanguinolent, independent of inflammation or local abrasion. Puriform effusion is of very rare occurrence, although it appears to have been observed. Dr. Hodgkin remarks, that "when the matter of the inflammatory effusion is of the most inorganizable kind, it has a very puriform appearance." We much question, however, whether the instances referred to are of the kind to which the author alludes; and, indeed, whether an effusion possessed of the characters of purulent matter occurs independent of the previous formation of adherent and organized false membranes, in which case, instead of considering it as an effusion of the most inorganizable kind, we should be disposed to regard it as a secretion of the reticulo-cellular tissue of which these membranes consist.

The occurrence of adhesions by means of false membranes of various extent and firmness has been well illustrated by Bichat. Dr. Hodgkin remarks, that cases in which the free surfaces of the membrane are so firmly united as to appear identified, the bond of union escaping observation, have been mistaken for the absolute deficiency of the pericardium. Instances in which such a mistake could occur must be of extreme rarity, as the adhesion is seldom found without such a degree of thickening as ought to lead to the detection of the true nature of the morbid change. The deposition of false membrane in shaggy, loose shreds upon the surface of the heart presents a very peculiar appearance; and Dr. Hodgkin thinks that it affords an explanation of "the idle and absurd stories of the heart having been found covered with hair." This shaggy form of false membrane is attributed to a considerable admixture of inorganizable matter in the product of the inflammatory process, by which adhesion is prevented from taking place. It may arise also, in part, from the extent of the effusion preventing the approximation of the walls sufficiently near to admit of early adhesion between them, at the same time that the agitation arising from the contractions of the heart, which under similar circumstances we have observed to be forcible, may exert some influence in the production of the peculiar form of the membranous, or rather flocculent or shred-like deposit. It is unnecessary to distinguish the many varieties which this loose membraniform deposit assumes on the surface of the heart and its external envelope; such as the reticulated appearance noticed by Corvisart, Laennec, and Bertin, and compared by these authors to the inner surface of the second stomach of the calf; or the undulated, transverse wrinkles described by Dr. Hope; but it may be observed, that these conditions probably indicate a still greater deficiency of organizable matter in the effused or secreted fluids, than the shaggy, shred-like form of deposition before mentioned.

The surface of the membrane is frequently found covered with a lymphous exudation of but little consistence, and, for the most part, readily detached; the membrane beneath being discoloured, of a yellowish or reddish tinge, and dull aspect. This exudation is usually of a pale yellow colour, sometimes with a greyish or greenish tinge, sometimes of a pale red. It is of soft curd-like consistence, and varies in thickness from one to two, and, in extreme cases, to three lines.

Thickening of the pericardium itself is probably of rare occurrence, and by some authorities the existence of this morbid change has been denied. Dr. Hope observes,* that "the pericardium is very rarely, if ever, thickened; that which is often regarded as thickening being nothing more than superimposed and intimately adherent false membranes." Dr. Hodgkin seems to speak with some hesitation upon this point. Morgagni, however, mentions a case, in which, beneath a whitish, ash-coloured exudation, there was thickening and slight redness of the membrane; and Broussais† relates one, in which, with a yellowish exudation on the surface of the heart, the serous membrane beneath was of a white

* Treatise on Diseases of the Heart.

† Histoire des Phlegmasies chroniques.

colour, and two lines in thickness, the subserous tissue being infiltrated with lymph.

The white spots so frequently found on the surface of the heart are thought by Dr. Hodgkin to depend, at least in by far the greater number of cases, on a deposit formed on the attached surface, contrary to the opinion of Baillie and Laennec, both of whom assert that they may be dissected off. Dr. Hope says, that they consist of condensed cellular tissue, and, with a little care, may generally be detached without injury to the pericardium beneath. An explanation is offered of the mode of their production, which is ingenious, though not to our minds satisfactory. That these spots are the product of some form of inflammation, we do not ourselves doubt; and, when we consider the obscurity of numerous cases of pericarditis, and (if we are to receive the views of M. Bouillaud,) the extreme frequency of occurrence of this disease, we need not be surprised at the circumstance of these spots being found where no symptoms during life had led to the suspicion of any affection of the pericardium. They appear to be analogous in many respects to the opaque and somewhat thickened patches which are found on the surface of other serous membranes, or in the cellular tissue beneath them, (for we believe them to occur in both situations,) and which are generally admitted to be the result of inflammation.

The results of inflammation of the serous membrane lining the thorax and reflected over the surface of the lungs, will be found detailed, partly in the second lecture, and partly in the fifth. The remarks in the second lecture, though applicable to serous membranes in general, have especial reference, as the author informs us, to the pleura. These, however, have already in part occupied our attention; it remains therefore only to notice the additional information on this part of the subject brought forward in the fifth lecture. The products of inflammation here, as in other membranes of this order, resolve themselves into changes taking place in the membrane itself, modifications of the natural secretions from its surface, the effusion or exhalation of portions of the circulating fluid, and the subsequent morbid processes arising out of the preceding.

The changes in the texture of the membrane itself are very simple, consisting of patches of redness of various shades, which, when closely examined, appear to consist of minute spots or stars, seated in the substance of the membrane, with striæ or vessels in the cellular tissue beneath. In some cases, however, there is diffused, uniform redness, of a pale colour, in patches, dotted with points of a deeper or brownish red. The morbid exhalations consist either of a fluid partaking of the character of the serum of the blood; of serum or serosity more or less charged with red particles, and hence assuming a sanguinolent appearance; or of lymphous exudations, giving rise to the deposition of layers of what has been termed albuminous deposit on the free surface of the membrane, and ultimately leading to the formation of false membranes. The changes in the secreted fluid present every variety, from a limpid, transparent, almost colourless secretion, little altered perhaps except in quantity, to sero-purulent or puriform matter, and mixed in every proportion with serous, lymphous, or sanguinolent exhalations. The secondary products of these states are false membranes on the pleural surfaces, becoming the medium of adhesions between them, more or less firm; in recent cases being soft, opaque, loose, of considerable thickness, and of various shades of yellow, pale citron, grey, or reddish colours; in those of longer duration, consisting of cellular or reticular tissue, thin, transparent, and colourless, frequently short and close in its structure, sometimes forming long bands or bridles, or of thicker, white, opaque, dense, almost cartilaginous firmness and texture. The varieties of membranous exudation and of false membranes are described by Laennec* with great minuteness. M. Gendrin gives the following summary of their characters:—1. Layer of coagulable matter of a greyish white colour, muco-purulent or gelatiniform, penetrated with serosity or with pus, and entirely amorphous. 2. A denser matter, much infiltrated with serosity, extensible, elastic, capable of being detached without breaking. 3. A friable substance, whitish like the white of egg, having occasionally a semi-cartilaginous consistence. 4. A more porous tissue, of less density and infiltrated with serous fluid of a reddish colour, in which vessels may be distinguished. This is sometimes infiltrated with blood, is somewhat more extensive than the healthy pleura, and approaches to cellular tissue in a state of chronic inflammation.†

The subject of Empyema occupies a considerable portion of this lecture. Dr. Hodgkin very properly limits the term to a collection of puriform fluid within the sac of the pleura, although, as he observes, it has been erroneously used with a much wider range of signification. Fluids of this description are found to vary much in their qualities, being sometimes a thin serosity, rendered yellowish, turbid, and opaque, by the admixture of numerous concrete particles; sometimes, especially in cases in which the collection is found in cysts, or rather pouches, formed by the false membranes, a yellowish fluid, of creamy consistence, presenting all the characters of true pus. It may be doubted, however, whether effusions presenting this character are altogether similar to the sero-purulent fluids before mentioned, since it is not improbable that, being the production of a secreting process going on in a different structure, (that is, in the reticular cells of the false membranes,—which, as we have already seen, have much affinity with the cellular or filamentous tissue,—rather than from the surface of the serous membrane itself,) they may differ on this account, as well as from their being more perfectly concocted, as Dr. Hodgkin thinks, by remaining long in contact with the secreting surface.

The excellent account given of the character and formation of these puriform effusions is followed by a condensed statement of the pathognomonic symptoms of empyema, and a clear exhibition of the natural processes of cure, by gradual absorption of the effused fluid and contraction of the walls of the thorax, and by evacuation of the fluid by means of a spontaneous external opening. The operation of paracentesis thoracis is then considered, and the best mode of effecting it,—whether by the knife or by cautery: to the latter method, except in cases threatening immediate suffocation, Dr. Hodgkin, we think with reason, gives the preference. An account of the manner in which the fluid is occasionally evacuated into the bronchi follows, and a very interesting case of this description is inserted, for which the author acknowledges himself

* Auscultation Mediate, vol.ii.

† Historie Anatomique des Inflammations, vol. i. p. 213.

indebted to M. Foville. Some of these details do not, perhaps, strictly fall within the professed object of the work before us; but we cannot regret that Dr. Hodgkin has availed himself of the opportunity of laying them before the public.

Cases in which a communication is established between the pleural sac and the bronchi and the puriform fluid brought up in large quantities by the mouth, though not frequent, are yet sufficiently so to fall under the observation of most of those who have extensive opportunities of seeing disease. Dr. Hodgkin, in addition to the one which he narrates on the authority of M. Foville, refers to one, if not more, which he saw in the clinical wards of the Edinburgh Infirmary. We have ourselves had occasion to see cases of this description. Laennec, Andral, Gendrin, and Louis report several instances; and the reader will find others in the writings of De Haen and many of the older authors, disguised under various names. It is the escape of the respired air through communications formed between the pleural sac and the bronchi, which gives rise to the most common form of pneumothorax; although air may be collected in the pleura independently of the existence of any such communication, originating, it is presumed, in the decomposition of the sero-purulent effusion. The value of the stethoscope, both in this affection and in empyema, is well pointed out by Dr. Hodgkin. Air may be produced from the decomposition of the sero-purulent effusion in the other serous cavities as well as in the pleura, and has been thought also, by Laennec and others, to be secreted or exhaled in certain states of the serous membranes. The presence of air in a serous sac from this last cause, however, we believe to be of exceedingly rare occurrence, if indeed it is ever found to exist. The evident liability to mistake the cadaveric production of gas, (which, as Dr. Hodgkin observes, in some states of the body takes place very shortly after death,) for an exhalation during life, must not be lost sight of in estimating the value of this alleged morbid phenomenon.

We extract the following passage respecting a peculiar form of the membranous deposit, which Dr. Hodgkin describes as presenting a worm-eaten appearance. The explanation of its formation, however, does not appear to us satisfactory, though we freely admit that we are unable to substitute a better.

"There is an appearance which deserves particular attention, and is sometimes met with in the pleura and other serous membranes, when inflammation has been of a chronic character, and its product of that mixed description, which, though it never assumes the form of cellular membrane, nevertheless becomes a permanent tissue, having a close, compact, and almost semi-cartilaginous character, resembling what Laennec has described as white non-fibrous tissue. The peculiar appearance to which I allude, consists in the surface of this dense adventitious structure being rendered uneven by irregular yet rounded depressions, sometimes distinct, sometimes running into each other like confluent small-pox, and presenting an appearance which suggests the idea of the part affected having been worm-eaten."

Dr. Hodgkin felt at a loss how to explain this appearance, when a case occurred, which, he thinks, throws important light on the subject and clears up the difficulty.

"In this case," he continues, "the pleura, thickened by dense semi-cartilaginous deposit, presented the worm-eaten appearance which I have mentioned; but the depressions, instead of being vacant, were filled with a soft, yet concrete inorganizable material, which was not however involved in, or adherent to, the more plastic form of the product of inflammation, as in some cases in which we have seen tuberculous or inorganizable matter shut up in the false membrane: on the contrary, this inorganizable matter was easily removed, leaving a clean and defined margin and surface to the depressions which it had occupied. Hence it would appear, that the worm-eaten appearance of the thickened surface is not produced by ulceration or other destruction of the thickening deposit, but that its deposition or formation had been prevented by the presence of a different material." (P. 124.)

The succeeding observations upon partial pleurisies and upon what have been termed dry pleurisies, that is, the formation of false membranes without any accompanying serous exhalation, we must pass over, contenting ourselves with referring to the remarks of Laennec* upon the latter of these subjects, and to the work before us for much useful information upon the former, more especially upon that part of it which relates to interlobular pleurisy with or without puriform collections. No satisfactory explanation is given, or perhaps in the present state of our knowledge can be given, of the occasional presence of detached bodies of a cartilaginous or fibro-cartilaginous nature in the pleura. The suggestion thrown out, as to the part which the processes of endosmosis and exosmosis may perform in the production of such bodies from soft coagula, appears to us scarcely justified even by analogy. The brief notice of atonic hydrothorax need not detain us, as we believe there are few now to be found in the ranks of the profession, who are not entirely in accordance with the author as to the extreme rarity of such cases.

The effects of inflammation in the subserous tissue are not often observed; Dr. Hodgkin, however, remarks that there is "sometimes a partial deposit, producing a slight thickening of a nearly opaque white colour, in some instances accompanied with a little puckering. The polished surface of the pleura at these spots is often quite natural; yet the occasional occurrence of a slight adhesion to the opposite surface of the pleura costalis must, I think, be allowed to indicate their inflammatory origin," (p. 134.) It is worthy of notice that these patches of opaque, white, thickened deposit bear considerable analogy to patches of a similar description occurring in the pia mater immediately beneath and almost inseparable from the arachnoid, and this fact may possibly tend to throw some light upon the white spots of the surface of the heart. If at all similar in their nature, they certainly favour the opinion of Corvisart advocated by Dr. Hodgkin, as to the situation of these last being upon the attached, and not upon the free surface of the serous covering of the heart.

The general changes induced by inflammation in the serous membrane lining the cavity of the abdomen and in its secretion, and the various effusions and exudations resulting, differ but little from those exhibited by other serous membranes. Still, there is considerable variety in the external forms and characters assumed by the false membranous deposits, in consequence of the extent of surface affected, the various nature of the subjacent organs, the greater laxity or closeness of the interposed cellular tissue, and the disturbance given to the diseased process by the facility of motion of the parts lying within the abdomen amongst each other. In the thorax, although there is a continual motion resulting from the alternate expansion and contraction of its capacity in the pro-

* Traité de l'Auscultation Médiate, vol. ii.

cess of respiration, this motion is confined within certain defined limits and is performed with a certain regularity, and its influence upon the membranous exudations is modified by the exhalation of fluid into the cavity of the pleural sac. The same remarks apply with even greater force to the serous envelope of the heart, while in the arachnoid any disturbance of the regular progress of diseased action from this cause must be almost inappreciable. In the abdomen, however, in addition to the regular peristaltic action of the several parts of the intestinal canal, the hollow viscera are continually varying in their state of distention from the passage of solid, liquid, or gaseous matters; are constantly changing tneir relative position in the performance of their respective functions; and are at the same time liable to be disturbed from their state of comparative repose by every change in the position of the body; the motions induced in each of these cases being also irregular and uncertain, exhibiting no analogy with the periodical and limited movements of respiration and pulsation.

The effects of inflammation upon the stricture of the peritoneum itself are: redness in patches, varying in tint and shade from a pale rose colour to a deep brown red, sometimes uniformly diffused, sometimes punctuated with minute points of a deeper shade; softening and pulpiness of its texture; thickening; and opacity; in which case the colour is whitish, and the aspect dull. The effusion also presents precisely similar characters to those found in other serous cavities. It is in the modifications exhibited by the exudations on the surface of the membrane, that we find the chief diversity in the products of inflammation to exist. Occasionally, the coagulable matter is thrown out in large quantity, which at the same time that it agglutinates the intestines together, forms an even surface towards the abdomen, and not being attached, or at least only partially so, and towards its margin, to the peritoneum lining the abdominal surface, assumes the appearance of false parietes. This peculiar appearance is certainly unusual, but we think Dr. Hodgkin is mistaken in supposing that it has not been noticed by others. Morgagni appears to allude to it, and relates an example which, as well as another mentioned by Mead, is quoted by Gendrin in his work on Inflammations.

A very interesting case is reported by the author, at page 140, which gives rise to the following observations:

"It seems to be essential to this result of peritonitis, that there should be a considerable quantity of the coagulable matter effused at once. When it is in smaller quantity, forming bridles of greater or less thickness and extent, these bridles are not merely found passing from convolution to convolution, but they also become attached to the parietes. This difference between the two cases is well worthy of attention. Were the extensive surface, which the false membrane presents in the one case, to become adherent to the parietes, their motion, so essential to the function of respiration, would be materially interfered with. In the other case, this effect is not to be apprehended from the partial adhesions in the form of bridles." (P. 142.)

We are not sure that we quite understand the author here. If it be intended to imply, that when the adhesions between the convolutions are of a more loose description and less uniform extent of membranous surface the parietes are always implicated, we must observe that we have upon several occasions seen extensive and intricate matting together of the intestines by layers and bridles of lymphy exudation, without that appearance of false parietes which we imagine Dr. Hodgkin intended to describe, and of which the cases mentioned by Morgagni and Mead, as well as the one related by himself, afford good examples. Yet in the examples to which we refer, the serous lining of the abdominal parietes had been so slightly implicated, as to give rise to no bridles of adhesion between it and the membranous exudation, agglutinating the intestines and other viscera together, or at least to so slight and partial an appearance of them as to permit of their being altogether disregarded. Indeed, we should say, that in cases of peritonitis terminating after an interval of a few days, this matted appearance of the intestines from the exudation of lymph confined chiefly to that part of the serous membrane which covers them, is the most common appearance observed, the inflammation rarely implicating the abdominal parietes, and its effects seldom being visible therein to any extent, unless in examples of perforation of some part of the intestinal tube, with escape of its contents into the peritoneal cavity, of rupture of the bladder, or of lesions from without,—the result of surgical operations, or of injuries accidentally or designedly inflicted.

The occurrence of collections of puriform matter is by no means infrequent in the abdomen.

"These collections, which vary in size from that of a pea to that of an orange, take place in those situations in which the concrete part of the inflammatory effusion is most considerable: hence they are most frequently met with in the lateral and angular parts of the abdomen, between the convolutions, and along the thicker edges of false membranes. They may be formed in cases in which the peritonitis appears to have been productive of an effusion, at first, of a very plastic character. These purulent collections are attended with ulcerative absorption; first, of that part of the serous membrane with which they are in contact, and afterwards of the parts subjacent to it. Now, if any accident tear through the feebly-organized exterior of the matter uniting the convolutions between which such collections exist, we have an opening into the intestine, formed from without, inwards, and effecting a communication between the purulent collection and the intestinal canal." (P. 143.)

We do not mean to question that this account is not to a certain extent correct, and that in many of these cases the process *ab initio* is as Dr. Hodgkin states; but we believe that the larger proportion of these partial puriform collections will be found to have a different origin, and to advance in an opposite direction to that which is here stated. Our own experience leads us to infer, that it is in the more confined situations of the membrane, and immediately in connexion with some local irritation, that these collections are chiefly found to occur; but the most frequent situations are not the lateral and angular parts of the abdomen indiscriminately, but the folds of the peritoneum between the rectum and bladder in the male, and between the rectum, uterus, and bladder, in the female, and in the vicinity of the cæcum in both; and, for the most part, we believe, in connexion with disease previously existing in the organs referred to. In instances of this kind there are adhesions where the edges of the folds of the peritoneum are in contact; and in the circumscribed pouch or sac thus formed, the puriform collection exists. The case of Harriet Polton related by the author is itself confirmatory of these views, and we could refer to many others in which there has been disease existing in the rectum, uterus, or caput cæcum, in connexion with sacculated puriform collections in their immediate vicinity, for further illustration, were it necessary. In Dr. Hodgkin's case, communications formed between the sac containing the puriform collection, and various parts of the intestines in immediate contact with it, and there had been effusion of their fluid contents into the sac; we have known similar instances in which effusion of the contents of the bowels took place into the peritoneum, and were confined within a circumscribed cavity formed, in part of the folds of the peritoneum, in part of the adhesions consequent upon the resulting inflammation. It is scarcely necessary to remark, that the mode of treatment proposed by Dr. Graves of Dublin, in cases of perforation of coats of the bowels, is founded upon the tendency which there exists to the rapid effusion of coagulable lymph between different portions of the peritoneum lying in contact, and thus forming, as it were, a close and circumscribed sac limiting the extent of the subsequent diseased actions. It is due to Dr. Hodgkin to state that when he treats of partial peritonitis in a subsequent part of the work, he seems to be fully aware of the frequent occurrence of such cases connected with, and probably arising from diseased states of the subjacent viscera, although, as it seems to us, he underrates their importance as a cause of the purulent collections found in cases of general peritonitis.

The tuberculated appearance assumed by the false membranes in cases of chronic peritonitis, arising, as Dr. Hodgkin thinks, from the admixture of inorganizable matter in considerable quantity with the lymphous exudation, has not been sufficiently studied. Many of the reported cases of tuberculated peritoneum, we have no doubt, are merely instances of chronic inflammation, attended with irregularity in the deposition of the coagulable matter of which the false membranes consist; but we are by no means disposed to agree with M. Louis that all cases of chronic peritonitis are tubercular, or in other words assume the characters of what has been too indiscriminately termed tuberculated accretions of the peritoneum. We look upon by far the greater number of cases of ascites to be inflammatory in their origin, and yet, few of these, although the effusion is frequently found turbid, and various parts of the serous membrane occupied with patches and flakes of lymph, are accompanied by tubercular accretions. There is no doubt, as Dr. Hodgkin justly observes, that chronic peritonitis is very frequently conjoined with tubercles; but we are much more inclined to attribute the accompanying inflammation, in such cases, to the irritation of the tubercular matter deposited on the serous membrane, whether in the form of layers, of accretions, or infiltrated into its tissue and the subserous tissue beneath, than to suppose that the tubercular disease is a necessary consequence of the more chronic form of inflammation established in the peritoneum. In short, we cannot but come to the same conclusion in regard to the connexion of scrofulous tubercles in the peritoneum with the process of inflammation, that Dr. Hodgkin has himself arrived at with respect to malignant tubercles.

"Scrofulous tubercles of various appearances and different sizes are not the only adventitious productions met with in conjunction with peritonitis. Scirrhous and fungoid masses and tubercles connected with the peritoneum, are found accompanied with more or less abundant effusion, and with bridles and films of adhesion, which sufficiently attest the coexistence of chronic inflammation with these growths. In such cases, however, the peritonitis may be regarded as a secondary affection." (P. 149.)

The appearances observed in those forms of peritonitis in which the product of the inflammation consists, either wholly or chiefly, of inorganizable matter, are next described. In these cases there is abundant effusion of a sero-purulent or more purely serous character, with but little tendency to the deposition of lymph on the surface of the serous membranes, or to the formation of adhesions. The sero-purulent form of effusion is more common, Dr. Hodgkin thinks, in injuries or disease of the pelvic viscera, and it is a peritonitis of this kind which most commonly occurs in the puerperal state. Dr. Hodgkin states that he has "repeatedly met with cases which appeared to bear a very close analogy to puerperal peritonitis, in males labouring under disease in or about the bladder," (p. 151.) It is when the effusion is in considerable quantity, and of a more purely serous character, being transparent, and clear, and containing little organizable matter, that the disease is classified as ascites, and the effusion considered to be of the atonic kind rather than of inflammatory origin. Dr. Hodgkin, at the same time that he acknowledges the difficulty, follows Dugés in attempting to discriminate between these two forms of disease, namely, inflammatory effusion of serum into the cavity of the abdomen, a result and form of chronic peritonitis, and the purely hydropic or atonic effusion, to which some authors are desirous of limiting the term ascites. Upon this point Dr. Hodgkin makes the following observations.

"It is difficult, even on inspection after death, to draw, in all cases, the line of demarcation; for, as I have remarked with respect to effusions into the pericardium and the pleura, so also in the peritoneum, the atonic and those of an inflammatory character pass insensibly into each other. The small quantity of tender false membranes which we so frequently meet with when the serous effusion is very copious, although they must unquestionably be referred to an inflammatory origin, are, in many cases, to be regarded rather as the result of a secondary action, which the presence of the fluid has excited in the peritoneum, than as indicating that the effusion was originally of an inflammatory nature. In those cases in which the peritoneal effusion is to be attributed to chronic peritonitis, a false membrane, which is often of great tenuity, but closely adherent to the serous membrane, may be detected wholly, or to a considerable extent, investing the surface of the peritoneum. The omentum is contracted, or corrugated and folded up, under the greater curvature of the stomach; and at times reduced to so small a compass, as to be scarcely recognizable. If old adhesions have partially fixed the omentum to some part of the pelvis, which is particularly frequent in females, in whom the adhesion of the omentum to the uterus probably takes place during pregnancy; the omentum, instead of being drawn up under the stomach, is found extended, in the form of a cord, between the stomach, and the part of the pelvis to which the adhesion had been formed. Besides the contraction of the omentum, the mesentery is found more or less shortened, by which the intestines are drawn up to the spine; and, if a hernia had existed, it will sometimes be found to have been completely reduced. The intestines are more frequently reduced in length than contracted in their caliber. In extreme cases, they probably lose nearly or quite half their dimensions, and the valvulæ conniventes are consequently placed very closely to each other. This contraction of the omentum, mesentery, and intestinal canal, is, I believe, perfectly analogous to the contractions of the chest which I have noticed as occurring after pleurisies; and seems to depend on the contractions which newly-formed parts undergo after they have become organized or permanent, as we see in the large cicatrices of extensive burns." (P. 151.)

We have extracted this passage because we believe that it contains nearly all that can be said upon the subject. Such morbid appearances as the author describes, it will not for a moment be doubted, must be the results of inflammation; but it is by no means so certain, that the very copious serous effusions coexisting with films of false membrane are of a purely atonic nature. To ascribe these films of false membrane to a secondary action, excited in the serous membrane by the presence of a fluid which is said to differ from the natural secretion of the part only in its greater abundance, is cutting the Gordian knot, but by no means unravelling the mystery, if indeed there be any mystery in the subject more than the prejudices of previously acquired notions have invested it with.

After noticing partial inflammations of various parts of the peritoneum, a honeycomb appearance similar to that of the pleura, and some other forms of inflammatory product of less importance, Dr. Hodgkin proceeds to describe the effects of inflammation in the subserous tissue. It may be observed, generally, that the effects of inflammation in the subserous tissue of the peritoneum are the same as in the corresponding tissue of the arachnoid, pericardium, and pleura, modified by the greater laxity of texture, and by the peculiarities of the several viscera between which and the serous tissue it lies interposed. We regret that we are compelled to pass almost without notice the valuable remarks of Dr. Hodgkin upon this part of the subject; we must, however, record our dissent from the opinion expressed by the author, that œdema of this tissue is probably less often the result of inflammation than of a more purely hydropical tendency, induced by disease of the lungs, heart, liver, or kidnies. The interesting details connected with the lacerability of this tissue are well worthy of attention, the separation of large portions of the inner coats of the intestines presenting a very remarkable appearance. We remember to have witnessed the dissection of a case of chronic peritonitis, in which the intestines were agglutinated together by membranous exudations, when several feet of the inner coats of the bowels were withdrawn continuously, with very slight effort, from the serous membrane and the matted tuberculiform deposit by which its different portions were inseparably united together. We cannot for a moment hesitate to ascribe this lacerability of the reticulated tissue, forming the bond of union between the serous, muscular, and mucous coats of the bowels, to the effects of inflammation in that tissue.

Ulceration of the serous membranes seems to be a very rare occurrence, and, although it may possibly take place in those cases in which, from ulcerative absorption in the subjacent organs, a breach of continuity ultimately arises in the serous membrane itself, it is by no means certain that this piercing of the membrane may not, in many instances at least, owe its origin rather to simple rupture from distention and pressure, than to any ulcerative action set up in the serous tissue itself. This would seem to be the case especially in perforations in the intestinal canal, and it must have occurred to those who have had much opportunity of inspecting diseased appearances, to have noticed the frequent instances of ulceration proceeding through the mucous and muscular tissues, and abrading and eroding these tissues for a considerable extent, at the same time that the serous tissue beneath seemed to resist the ulcerative action, and, retaining its usual appearance at the bottom of the ulcers, formed a line of demarcation between the diseased inner coats of the bowels and the general cavity of the peritoneum. This explanation will scarcely account for the perforations occasionally taking place in the pleura, where a communication is formed between the bronchi and those partial collections of puriform matter found in interlobular pleurisy. Instances of ulceration of the arachnoid, dura mater, pericardium, and peritoneum are recorded by various authors, but Dr. Hodgkin makes no mention of cases of this description as having fallen under his notice; and, indeed, the only references he makes to the subject of ulceration connected with the serous membrane, are a few incidental remarks upon the occurrence of ulcerative absorption in the process of what has been termed empyema of necessity, and in perforation of the intestines.

On the subject of gangrene Dr. Hodgkin is more explicit, although he is of opinion, that, independently of the existence of this change in adjoining structures, or of accidental injury, it is of extremely rare occurrence.

"Gangrene is an extremely rare termination of inflammation in any of the serous membranes, especially of idiopathic inflammation. Lieutand has mentioned one instance in the pericardium, and the highly offensive and discoloured condition of effusions into some of the serous cavities, described by Bichat, was probably attended with a state of membrane approaching to sphacelus. I cannot say that I have ever seen an unequivocal instance of gangrene of a serous membrane, except when it was either propagated from the adjoining structures, or the effect of accidental injury. We have an example of the first of these in gangrene of the lung; and of the second in strangulated hernia. It is by no means uncommon to find both the serous membranes, and the false membranes formed upon them, of a very dark colour; but this is of a different nature from gangrene, and is to be carefully distinguished from it. It depends on an altered condition of the blood in the minute vessels; and is apparently most frequent in situations in which inflammation or irritation had at one time existed, but had subsequently subsided. Or it may be decidedly cadaveric, and depend on altered blood, either in its vessels, or extravasated, or transuded." (P. 61.)

Dr. Hodgkin subsequently refers to a case of gangrene of the pleura occurring in Guy's Hospital, in which the extent of the disease was very considerable, and, though accompanied by gangrene of the lung, did not appear to arise solely from this cause. It is, no doubt, a rare occurrence for the serous membranes to be attacked with sphacelus, and when it is considered that, independently of the cellular tissue seated beneath them, they can for the most part be but little liable even to inflammation, we shall not expect to find this change without a corresponding change in the neighbouring organs. When, however, it does occur, the serous tissue becomes of a greyish-black colour, is softened, and breaks down under the finger into a loose fetid putrilage. Dr. Carswell considers the lacerability of the subserous tissue of the intestines before noticed as owing to a loss of vitality, and consequently to a state approaching to sphacelus in this texture; and in such cases, assuming the opinion to be correct, the serous coat itself must in a measure partake in the same morbid process. Accordingly, in some instances it very readily gives way to a slight force. This appears to have occurred in the case of Elizabeth Sayce, reported by Dr. Hodgkin as an example of this lacerability of the subserous tissue. "In attempting to separate the convolutions of the intestines, the adventitious matter which united them, and also the muscular and peritoneal coats, gave way, and even torn through; leaving entire the mucous coat, which adhered so slightly to the muscular, that several feet of it could be withdrawn without the slightest difficulty." (P. 170.)

Osseous deposits connected with the serous membranes, are commonly found in circumscribed continuous patches, or sometimes sprinkled in the form of minute spiculæ, beneath the surface of the membrane; the patches are of various extent and configuration, but usually thin, condensed, and amorphous in their structure. They are rarely found beneath the arachnoid. Dr. Baillie, however, refers to an example as existing in the collection of Soemmering; and Dr. Hodgkin mentions that a specimen is preserved in the collection of Guy's Hospital, in which a few small particles of bony matter were found in this situation. Bony matter is not unfrequently deposited beneath the pericardium in the form of thin plates. Baillie mentions an instance in which the deposition of ossific matter had been so extensive as to spread over a considerable portion of the pericardium, and Dr. Hodgkin refers to a preparation in which an osseous plate occupies a large portion of the base of the heart, where it forms a complete bony ring. Osseous deposit is also found occasionally on the attached surface of the pleura. Dr. Hodgkin mentions a remarkable instance occurring in an old man, in which there was not merely a plate of bone beneath the costal pleura nearly half encircling the chest, but also a considerable mass of the same substance. These deposits are evidently regarded by Dr. Hodgkin as the result of inflammation.

The deposition of tubercular matter is very frequently found in connexion with serous membranes, although we must be careful not to be led into the error of supposing that every case which has been described by authors as tubercular is really an instance of this form of disease. Dr. Bacon in his work on Tuberculated Accretions of the Peritoneum, as Dr. Hodgkin remarks, appears to have included under that term, instances of chronic inflammation of the peritoneum, having no connexion with the scrofulous deposit to which it is desirable the term tubercular should be especially limited. It is probable, that genuine tubercle is seldom found on the free surface of the serous membranes, except in cases where there has been a previous deposit, the result of inflammation; but we are by no means inclined to suppose from the occurrence of tubercular matter in false membranes, that the inflammation has had any share in its deposition in such situations, further than through the general effects which may have been produced, either by the inflammation or the treatment necessary to subdue it, tending to favour the development of the tubercular constitution. The tubercles in their simple and uncomplicated state are usually found in the subserous tissue, but, as Dr. Hodgkin correctly observes, collections of a scrofulous deposit, in connexion with serous membranes, are not confined to the cellular membrane on the attached surface.

"Besides these isolated collections we meet with other phenomena, both on the unattached surface and in the membrane itself. On the smooth unattached surface, collections of tuberculous or scrofulous matter, varying in size, number, and figure, are sometimes found dispersed through the substance of recent false membranes, the production of ordinary inflammation." . . . "There are other cases in which the whole of the adventitious deposit is so modified by the strumous diathesis, that, instead of the ordinary false membrane, we meet with a material known by the name of tuberculous, in the sense in which this word is used to designate the character of the material rather than its particular form." . . . "In an early stage of this deposit upon the unattached surface of a serous membrane it forms a concrete layer, possessing some degree of translucence, but wanting the lamellar form of the plastic product of inflammation, and rather exhibiting a peculiar shortness and friability. At a later stage, instead of a semi-translucent layer, we find an opaque substance, presenting some shade of yellowish white; in fact, assuming the character of what is called crude tuberculous matter, and generally coincident with other varieties of this deposit in other parts of the body." (P. 61, 62.)

Miliary tubercles, it is subsequently said, are sometimes to be met with, though rarely, in the substance of the serous tissue itself.

The most frequent situation of tubercular deposit in the serous membranes is in the peritoneum, either in the subserous tissue, in the substance of false membranes deposited on its surface, or modifying the inflammatory product in the manner which we have just alluded to, and of which we take the preceding quotation to be a description. The genuine tubercle is seldom met with but in the subserous tissue, as Dr. Hodgkin remarks, and of its occurrence in this situation he gives the following characteristic account.

"When they occur beneath the peritoneal coat of the intestines, which is by no means unfrequently the case, they are generally of small size, but very numerous; and, at times, give to the intestines the appearance of being sprinkled with particles of boiled rice. These little tubercles are often surrounded with a vascular areola, which sometimes acquires a dark colour, from the change of the blood within the vessels. When very minute, and meriting the appellation of miliary, they appear to be situated between the layers, into which the serous membrane itself, notwithstanding its tenuity, may sometimes be split." (P. 71.)

A case is reported by M. Louis in which "the omentum covered the greater part of the small intestine, forming a mass from twelve to fifteen lines thick, uneven, alternately yellow and bluish in colour, composed of the tuberculous and grey semi-transparent matter. The meso-colon and meso-rectum presented the same alteration, but were only half as thick as the omentum."*

In the arachnoid and in the pericardium tuberculous deposits are of very rare occurrence, though Dr. Baillie mentions having met with them in both these situations. M. Lombard has seen them in false membranous adhesions, formed between the heart and the pericardium.† Dr. Hodgkin speaks very doubtfully of their presence in the substance or on the free surface of the pleura, although he has seen the false membrane lining the cavity in a case of chronic pleurisy very closely sprinkled with small, opake, miliary tubercles, which appeared to be seated on the serous membrane itself. Laennec mentions the occurrence of tubercles developed at the surface of the pleura under similar circumstances. We are much disposed to agree with the opinion expressed by Dr. Hodgkin upon this point; at the same time, we must observe, that some of the highest authorities are very decided as to the occurrence of tubercular matter, both in the substance of the pulmonary serous membrane and upon its unattached surface. Corvisart and Laennec both mention the exudation of tuberculous matter from the surface of the pleura, and the latter author refers to a case reported by Boerhaave which he considers to have been of this nature, and to another observed by M. Recamier; and also reports at length a case of M. Cayol's, in which "the right lung appeared absolutely transformed into a tuberculous mass; but, on examining it more

* Pathological Researches on Phthisis.

† Treatise on Diseases of the Heart, by Dr. Hope, p. 608.

attentively, it appeared that this matter was contained in the cavity of the pleura itself, which it entirely filled. It was a mass of a carious consistence, in which no distinct tubercle was distinguished. The thickness of this mass was about two fingers' breadth on the anterior and posterior parts of the lung, and a little less at the side."* It obviously admits of question whether the preceding cases are not rather instances of the products of inflammation, modified by the circumstances under which they occurred, than of purely tuberculous deposition. It is stated by Dr. Carswell, that "the formation and subsequent diffusion of tuberculous matter is also observed on the secreting surface of serous membranes, particularly the pleura and peritoneum;" but, from the illustrative plate referred to† and the accompanying description, the tubercles appear to have been deposited in false membranes, formed on the surface, rather than in the serous tissue itself.

We have now examined in detail the principal morbid changes observed in the serous membranes, with the exception of those which will find a more appropriate place, when we come to consider the subjects comprised in the remaining portion of the volume, viz. parasitic animals, malignant adventitious structures, and the indications afforded by colour. We must however defer to a future occasion the examination of these subjects, as the important nature of what has here engaged our attention has led us to exceed the limits within which we wished to have confined our observations.

It is hardly necessary, after the trouble we have taken to lay before our readers a full account of the contents of Dr. Hodgkin's work, to give here our formal opinion of its merits. No book but a good one could have claimed from us so much time and attention as we have bestowed on it, or have supplied or suggested materials to fill so many of our pages as we have devoted to its consideration. It is, however, scarcely doing justice to our judgment to say that the work is simply good: it is in every respect an excellent production; calculated to advance the progress of pathological science, and destined to take a permanent place among the higher order of the medical classics of this and other countries.

---

### Art. III.

* Traité de l'Auscultation Mediate, Vol. II.

† Fasc. I. Tubercle, t. 5. f. 4.

